# Endometriosis Among Surgical Specimens in Gabon: A 35-Year Retrospective Study

**DOI:** 10.1089/whr.2023.0154

**Published:** 2025-02-11

**Authors:** Irène Pegha-Moukandja, Sydney Maghendji-Nzondo, Jean Engohang-Ndong, Sidonie Nguindzi-Ogoula, Euloge Ibinga, Gabrielle Atsame-Ebang, Géremy Abdoul Koumbadinga, Ophilia Makoyo, Ludjer Mpiga-Ekambo, Jean Bernard Lekana-Douki, Edgard Brice Ngoungou

**Affiliations:** ^1^ Centre International de Recherches Médicales de Franceville, Franceville, Gabon.; ^2^ Université des Sciences de la Santé de Libreville, Libreville, Gabon.; ^3^ University Dr. NE, New Philadelphia, Philadelphia, Ohio, USA.; ^4^ Centre Hospitalier Universitaire Mère-enfant de Libreville/Gabon, Libreville, Gabon.; ^5^ Centre Hospitalier Universitaire de Libreville, Libreville, Gabon.

**Keywords:** endometriosis, Gabon, gynecological diseases, infertility, epidemiology

## Abstract

**Background:**

Endometriosis is a chronic inflammatory gynecological disease affecting many women worldwide. In Gabon, data on this disease has never been published. Created in 1978, the laboratory of the Department of Pathological Anatomy at the University of Health Sciences was the only laboratory for histological analysis throughout all the country until 2009.

**Methods:**

A descriptive and analytical retrospective study was conducted using data from the medical records of women whose operation specimen samples were examined between 1987 and 2022.

**Results:**

A total of 6666 files were collected for the period between 1987 and 2022. The mean age of the patients was 38.0 ± 10.8 years. The proportion of endometriosis between 1987 and 2022 was 7.3% [6.7–7.9]. The year 2004 had the highest prevalence (23.2%). Endometriosis was significantly elevated in patients aged 36 to 50 years (10.8%), followed by those aged 51 years and over (9.6%) (*p* < 0.001). The uterus was the most resected surgical part (40.3%) and the most affected organ (72.6%). Malignant (23.9%) and benign (51.4% including 29.6% leiomyomas) tumors were the most common pathologies. In the final model, age, in particular the groups of [35–50 years] (OR = 11.4 (95% CI [2.8–46.5]) and [51–89 years] (OR = 11.2 (95% CI [2.7–46.1]), salpingitis (OR = 2.6 (95% CI [2.1–3.3]), and benign tumors (OR = 1.3 (95% CI [1.1–1.6]) were risk factors for the occurrence of endometriosis.

**Conclusion:**

This study is the first published study, which reveals that endometriosis is a health issue in Gabon. In order to better characterize this pathology in the country, it would be wise to conduct prospective studies, including Knowlege Attitude and Pratice's (KAP) studies.

## Introduction

Endometriosis is a debilitating disease responsible for pelvic pains and infertility. It is characterized by chronic inflammation and impacts the quality of life.^1^ In an ectopic situation, this pathology is defined by the presence of endometrial tissue called “lesions.”^1^ The most common topographies for endometriosis lesions are the ovaries, ovarian fossa, fallopian tubes, uterosacral ligaments, and the posterior part of the Douglas cul-de-sac.^2^ Based on the published work, the proportions of endometriosis would vary between 2% and 15% in childbearing-age women and 30% to 50% in subfertile women^3,4^ These frequencies were recorded in American, European, Asian, and African countries where surveys were conducted in hospital settings.^5^ There are no published data for some countries, and this is the case for Gabon.

Diagnosis of endometriosis includes physical examination, medical imaging, and surgical visualization. Among medical imaging tests, pelvic or endovaginal ultrasounds can detect particular foci of endometriosis as they have good sensitivity for endometriomas.^5^ Nevertheless, these ultrasounds only explore the gynecological sphere. Increasingly, magnetic resonance imaging is being promoted as a non-invasive reference method for the diagnosis of endometriosis to explore all suspicious parts in a patient.^5–7^ However, the accessibility of this technique in several regions of Africa, such as Gabon, is still minimal, in addition to having limited sensitivity in detecting superficial lesions, its association with high cost, and the need for qualified staff.^8,9^

On the other hand, minimally invasive (laparoscopy) or invasive (laparotomy) surgical techniques dedicated to diagnosis allow direct visualization of endometriosis lesions and sampling of parts for histological confirmation.^5,6^ These techniques have often been practiced in Gabon for the diagnosis of endometriosis and served as a basis for our work. In addition, the histological examination makes the presence of endometriotic lesions indisputable^10^ by establishing the presence of an inflammation, a tumor, or other associated pathologies.

Although some advances are observed in diagnostic techniques for this disease, it remains enigmatic. The virtual absence of published data on endometriosis in Gabon led us to take stock of the health situation of this disease over a period of 35 years, from 1987 to 2022.

## Materials and Methods

### Study site and settings

The present study was carried out in the laboratory of the Department of Pathological Anatomy (LAP) at the University of Health Sciences (USS) (LAP/USS). The USS is located in the commune of Owendo in the city of Greater Libreville, south of the department of Komo-Mondah in the province of Estuaire. The LAP/USS was established in 1978. For 32 years (from 1978 to 2010), it was the only public laboratory in the country to receive histopathological samples.

### Type and period of study

A retrospective, descriptive, and analytical study was conducted using medical record data collected from January 1, 1987, to December 30, 2022.

### Study population

The study population included medical records of all women whose surgery parts were received at the LAP/USS during the study period.

Medical records of women who had gynecological and/or digestive and pelvic pathology requiring an excision or biopsy-excision were systematically included. In addition, all incomplete records with no histological diagnosis and all records of women with breast pathology were not included.

### Anatomopathological analyses

#### Organs of the pelvic region associated with this study

The organs associated with this study were all parts of the uterus, fallopian tubes, ovaries, peritoneum, appendix, Bartholin’s glands, pelvic ganglia, bladder, colon, small intestines, rectum, vagina, cervix, all parts of the vulva, and umbilicus.

The files were said to be considered completed when they contained the following information: the identity ID of the patient whose part was received, the date of receipt of the part, the age or not of the patient whose part was received, the obstetric history of the patient, the name of the structure, which sent the part for anatomopathological analyses, the prescriber of the examination, the examination requested, the clinical signs, which prompted the examination, the anatomical description of the type of part sent for analyses, the histopathological description of the lesions observed as well as their topographical locations.

#### Endometriosis case definition

Throughout this study, endometriosis was defined as any part of women in whom one or more lesions (endometrial-like tissue, glands and stroma, siderophages, *etc.*) were observed during histological analyses.

### Study process

The surgical specimens analyzed during this work were obtained from pelvic or abdominal surgery or both in patients who presented symptoms related or not to endometriosis.

The resected organs were those of the pelvis: uterus, fallopian tubes, ovaries, vagina, cervix, and vulva. There were also those of the abdomen: the peritoneum, the appendix, the colon, the small intestine, the rectum and the umbilicus. Histopathological analyses of these organs were carried out in the Pathological Anatomy laboratory in Libreville’s University of Health Sciences.

Data were collected from medical files archived in the Pathological Anatomy laboratory and entered into a database created for the study.

### Statistical analysis

Data were analyzed using Stata 14 software (College Station, TX, USA). The age variable was transformed into a categorical variable with four classes: [12–20] years, [21–35] years, [36–50] years, and [51–89] years. The year variable was also transformed into 4 classes: [1987–1996], [1997–2006], [2007–2016], and [2017–2022]. Each class/classification corresponded with a decade with the exception of the last one which corresponded to half a decade.

These pathologies were grouped into benign tumors (leiomyoma, condyloma, hydrosalpinx, and polyp) and malignant tumors (adenoma and carcinoma). The other pathologies (endometriosis, ectopic pregnancy [EUG], and salpingitis) remained independent. Qualitative variables were described using percentages. Women’s ages were described using the mean (±standard deviation) and the 95% confidence interval (CI).

Comparison of proportions was carried out using the Pearson Chi-square test. The one-way analysis of variance test was used to compare the average ages of women in different decades.

The Mantel–Haenszel Chi-square test was used to investigate the association between the occurrence of endometriosis and the clinical characteristics of women by estimating the odds ratio (OR) and its 95% CI. A stepwise descending logistic regression was carried out by integrating the independent variables having been associated with endometriosis in the bivariate analysis as well as the other independent variables whose degree of significance was between 0.05 and 0.25. The significance threshold for all analyses was set at α = 0.05.

## Results

### Sociodemographic characteristics of patients examined and treated between 1987 and 2022

A total of 6,666 medical files was recorded between 1987 and 2022. These were the files of patients who had tissue samples taken for histopathological analyses in the LAP/USS. Among them, 2611 patients (39.2%) were registered between 1987 and 1996, 1675 patients (25.2%) between 1997 and 2006, 1586 patients (23.8%) between 2007 and 2016, and 794 patients (11.9%) between 2017 and 2022.

Among the different surgical specimens received, the uterus was the most common part with 50.3% (3347), followed by the tubes 26.0% (1728), the ovaries 22.7% (1508), the appendages appendix 3.3% (219), and the other parts (cervix, vagina, vulva, anus, colon, small intestine, bladder, peritoneum, omentum, and gallbladder), which were 16.8% (1114). A gradual decline in the number of women was observed from one decade to another.

One hundred and twenty two (122) patients did not provide their age. Over the 35 years, the average age of the patients was 38.1 ± 11.4 years; 95% CI = [37.8–38.4 years].

The average age increased significantly (*p* < 0.0001) from one decade to another: 35.8 ± 10.6 years between 1987 and 1996, 38.4 ± 11.3 years between 1997 and 2006, 39.7 ± 11.3 years between 2007 and 2016, and 42.0 ± 12.7 years between 2017 and 2022.

The majority of women who received a pathological sample were those aged 21 to 35 years (41.4%; *n* = 2707) and those aged 36 to 50 years (41.4%; *n* = 2712). Women aged 51 and over represented 13.6% (892) and those aged 12 to 20 only 3.6% (233) of the population. Characteristics of patients studied over 3½ decades are detailed in Table 2.

### Proportion of endometriosis

**FIG. 1.**
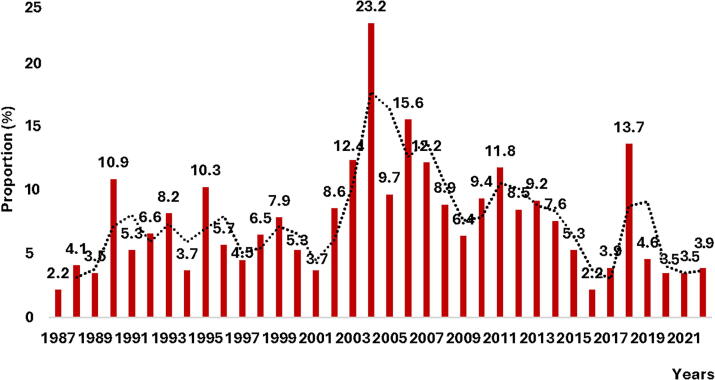
Annual prevalence of endometriosis from 1987 to 2022.

The prevalence of endometriosis between 1987 and 2022 was 7.3% (*n* = 486; 95% CI = [6.7–7.94]) and 5.5% of the population presented with adenomyosis. Figure 1 shows a sinusoidal pattern of the prevalence of endometriosis in the anatomo-pathological laboratory over 3½ decades. Peaks in the prevalence of endometriosis of 23.2% in 2004, and 15.6% in 2006 were observed (Fig. 1). The prevalence of endometriosis was 5.8% (151) between 1987 and 1996; it was 9.7% (162) between 1997 and 2006, 8.7% (138) between 2007 and 2016, and 4. 5% (35) between 2017 and 2022. A progressive increase in the pathology (endometriosis) has been observed over the years. The prevalence of endometriosis was significantly high in patients aged 36 to 50 years (10.8%; *n* = 294), followed by those aged 51 and over (9.6%; *n* = 86) (Table 2). The average age of women with endometriosis was 42.5 ± 8.9 years.

We also observed that the average age of women diagnosed with endometriosis increases significantly (*p* < 0.05) with each decade (Table 1).

**Table 1.** Relationship Between Age and Endometriosis and for Each Decade

Ages (years)	1987 to 1996			1997 to 2006			2007 to 2016			2017 to 2022		
	EMT (−) *n* = 2417	EMT (+) *n* = 147	*p*	EMT (−) *n* = 1478	EMT (+) *n* = 159	*p*	EMT (−) *n* = 1425	EMT (+) *n* = 136	*p*	EMT (−) *n* = 744	EMT (+) *n* = 35	*p*
Mean Age (±SD)	35.5 (±10.7)	41.3 (±8.7)	<0.0001	38.0 (±11.5)	41.4 (±8.9)	0.0004	39.2 (±11.4)	44.4 (±8.4)	<0.0001	41.8 (±12.8)	45.2 (±10.0)	0.12
Age group [*n* (%)]												
[12–20]	105 (100.0)	0 (0.0)	0.001	60 (96.8)	2 (3.2)	0.001	49 (100.0)	0 (0.0)	0.001	17 (100.0)	0 (0.0)	0.09
[21–35]	1271 (97.1)	38 (2.9)		594 (94.7)	33 (5.3)		507 (96.4)	19 (3.6)		240 (98.0)	5 (2.0)	
[36–50]	809 (90.2)	88 (9.8)		638 (86.3)	101 (13.7)		657 (88.4)	86 (11.6)		312 (94.3)	19 (5.7)	
[51–89]	232 (91.7)	21 (8.3)		186 (89.0)	23 (11.0)		212 (87.2)	31 (12.8)		175 (94.1)	11 (5.9)	

EMT: Endometriosis.

### Clinical characteristics of endometriosis

#### Topographic location of the endometriosis

**FIG. 2.**
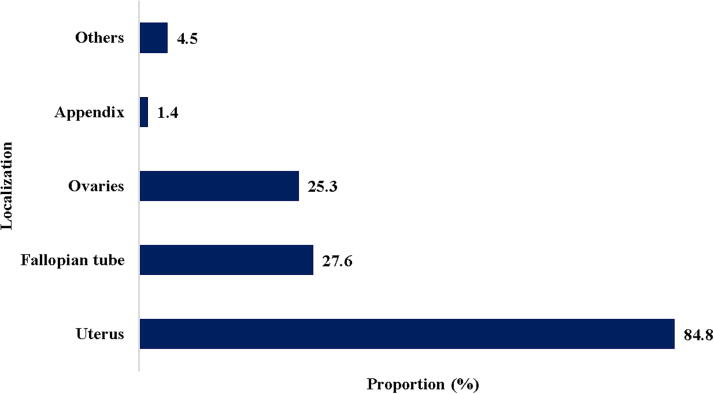
Localization of endometriosis. Others: peritoneum, small intestine, colon, anus, umbilicus, cervix, bladder, vagina, vulva, and epiploon.

Figure 2 shows the different topographic locations of endometriotic lesions. The uterus was the organ most often affected, with 84.8% (412), followed by the tubes with 27.6% (134), the ovaries 25.3% (126), and the other parts of the pelvic region with 4.5% (23).

#### Pathologies

**FIG. 3.**
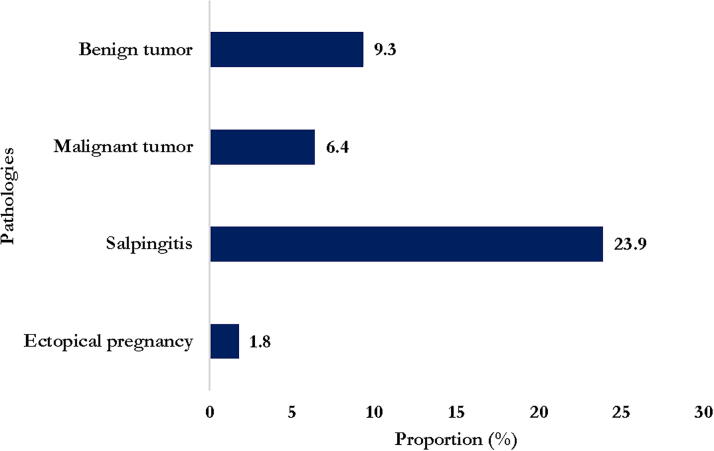
Pathologies observed in women with endometriosis.

During the study period, four pathologies and groups of pathologies other than endometriosis were observed. Benign tumors represented the most frequent pathologies, 39.0% (2599). Among these tumors, leiomyomas were 29.6% (1954). Salpingitis represented 15.0% (987) of the pathologies found, followed by malignant tumors at 10.7% (710) with 9.1% (609) of carcinomas. The ectopic pregnancy, for its part, represented 8.0% (529). Malignant and benign tumors, as well as salpingitis, were the most frequently observed pathologies (Figs. 2 and 3). These pathologies were diagnosed both in women affected and in those without endometriosis (Fig. 3; Table 2). In addition, Table 2 shows that salpingitis and benign tumors are more prevalent in women without endometriosis (*p* < 0.05). A significant decline in these pathologies over the years (between 1987 and 2022) was observed with the exception of benign and malignant tumors which increased (Fig. 3; Table 2).

**Table 2.** Pathologies Observed in Women with Endometriosis Versus Women Without Endometriosis by Decade

Pathologies	1987 to 1996		1997 to 2006		2007 to 2016		2017 to 2022	
	EMT (−) *n* = 1871	EMT (+) *n* = 145	EMT (−) *n* = 1048	EMT (+) *n* = 142	EMT (−) *n* = 967	EMT (+) *n* = 98	EMT (−) *n* = 519	EMT (+) *n* = 35
Ectopical pregancy	454 (99.1)	4 (0.9)	50 (94.3)	3 (5.7)	6 (85.7)	1 (14.3)	10 (90.9)	1 (9.1)
Salpingitis	495 (89.5)	58 (10.5)	256 (86.5)	40 (13.5)	88 (88.0)	12 (12.0)	32 (84.2)	6 (15.8)
Malignant Tumor	205 (94.5)	12 (5.5)	160 (88.4)	21 (11.6)	181 (95.8)	8 (4.2)	119 (96.8)	4 (3.2)
Benign tumor	717 (91.0)	71 (9.0)	582 (88.2)	78 (11.8)	692 (90.0)	77 (10.0)	358 (93.7)	24 (6.3)

EMT, Endometriosis.

#### Determination of association between pathologies and endometriosis

The initial model revealed that age ([21–35], [36–50], and [51–89] years) was associated with endometriosis (OR = 4.2 [*p* = 0.002]; OR = 14.0 [*p* < 0.0001]; OR = 12.3 [*p* < 0.0001]). A significant association between the occurrence of endometriosis, salpingitis, and benign tumors was demonstrated [OR = 1.9 (*p* < 0.0001) and OR = 1.7 (*p* < 0.0001)]. The odds ratio of women suffering from endometriosis were high in women with these pathologies (Table 3). Ectopic pregnancies were significantly associated with the occurrence of endometriosis. However, this association was in favor of protection against it (OR = 0.2; *p* < 0.0001). The final model retained the following characteristics: age, in particular the groups of [36–50] years and [51–89] years, salpingitis, and benign tumors as risk factors for the occurrence of endometriosis in women (Table 4).

**Table 3.** Association of Age and Lesions and Pathologies Observed and the Occurrence of Endometriosis

	Women without endometriosis *n* (%)	Women with endometriosis *n* (%)	Odds ratio [95% CI]	*p*
Age group (years)				
[12–20]	231 (99.1)	2 (0.9)	1	
[21–35]	2612 (96.5)	95 (3.5)	4.2 [1.02–17.1]	0.02
[36–50]	2418 (89.2)	294 (10.8)	14.0 [3.5–57.1]	<0.0001
[51–89]	806 (90.4)	86 (9.6)	12.3 [2.9–57.1]	<0.0001
Pathologies				
Ectopic pregnancy	520 (98.3)	9 (1.8)	0.2 [0.1–0.4]	<0.0001
salpingitis	871 (76.1)	116 (23.9)	1.9 [1.5–2.4]	<0.0001
Malignant tumors	665 (93.7)	45 (6.4)	0.8 [0.6–12]	0.3
Benign tumors	2349 (90.7)	250 (9.3)	1,7 [1.4–2.1]	<0.0001

**Table 4.** Final Model of Factors Associated with the Occurrence of Endometriosis

Pathologies	Odds ratio	[95% CI]	*p*
Tranche d’âge			
[12–20]	1		
[36–50]	11.4	[2.8–46.5]	0.001
[51–89]	11.2	[2.7–46.1]	0.001
Ectopic pregnancy			
No	1		
Yes	0.3	[0.1–0.6]	0.001
Salpingitis			
Non	1		
Oui	2.6	[2.1–3.3]	<0.001
begnin Tumors			
No	1		
Yes	1.3	[1.1–1.6]	0.008

## Discussion

This retrospective observational study with descriptive purposes provided, for the first time, the proportion of endometriosis in 6666 women from 1987 to 2022 (35 years).

From one decade to the next, the total number of women varied. This variation could be explained by the fact that since its establishment in 1978, the LAP/USS was, for 32 years (from 1978 to 2010), the only public laboratory for histological analyses to receive parts samples from all over the country. The opening of other structures caused a dispersion of cases of histological analyses. Another reason could be the behaviors, beliefs, and customs that women themselves may have. They all do not necessarily go to the hospital even if they have symptoms related to endometriosis. Indeed, in many cultures around the world, particularly in Africa, some people do not seek conventional medicine consultations.^11^ They’d rather resort to traditional healers or even to spiritual authorities (pastors and priests of churches, fetishists, or marabous). Another explanation would be that the USS sometimes experiences strikes, which lead to the temporary closure of laboratories within the university, including the LAP/USS discussed here.

In this study women aged 21 to 50 represented more than ¾. This observation is normal given that menstruation occurs between the ages of 12 and 16. Then, during the third decade (20–30 years) of their lives, women seek to procreate. In the fourth decade of female life (30–40 years), hospital visits are more frequent in cases of reproductive issues. Although those aged 21 to 50 were the most represented, a total of 13.6% (892) of our patients were still aged between 51 and 89 years. However, the onset age of menopause in many African countries is around 56 years.^12,13^ The endometriosis lesions in these women over 51 could have been accidental discoveries even if the inflammatory syndrome persists during menopause.

Over the 35 years of study, the proportion of endometriosis was 7.3%. This was higher than the prevalences found in Uganda in 2012,^14^ in South Africa,^15^ and in the South-East/South-West of Nigeria.^16,17^ However, this proportion in Gabon is almost similar to that reported in northern Nigeria in 1979,^18^ and lower than those described in Cameroon in 2007.^19^ Concerning the studies carried out in Uganda, South Africa, and southeast/west Nigeria, the average ages of the women were close to those of the women with endometriosis in our Gabonese study. On the other hand, the number of women in these studies was low (around 218 to 528) compared to the 6666 women in the Gabonese study. Our study focused on the surgical parts of women in all areas. The Cameroonian study, for its part, explores data from subfertile women when we know that subfertility is a factor strongly associated with endometriosis; it is therefore normal to have a high prevalence within such a population.

The prevalence of endometriosis was highest in patients aged 36 to 50 years (10.8%; *n* = 294), followed by those aged 51 and over (9.6%; *n* = 86). These results are similar to what has been commonly reported, namely that the diagnosis of this disease is made in women of advanced age.^20^ In addition, the average age of women with endometriosis was significantly higher than that of all women in this study. This significant increase could also imply that the diagnosis of endometriosis is made mainly in women of an older age.

However, the low proportion of endometriosis in women under 20 years old could be due to the reluctance of surgeons to perform laparotomies in young women except in cases of life-threatening emergencies. Added to this is the fact that under the age of 20, there are few outbreaks and lesions of endometriosis due to the fewer number of menstrual cycles in these girls.

The uterus was the most affected organ with 72.6% (172), followed by the tubes with 13.9% (33), the ovaries 7.6% (18), and other sites with 8.4% (20); In most women over 42, the main surgery was total hysterectomy. In a Cameroonian study of 441 patients who underwent a laparoscopic examination, the prevalence of endometriosis was 13.5%; the ovarian location was the most frequent and represented almost a third (35.79%) of the cohort;^21^ there was no details regarding lesions within the uterine muscle (adenomyosis), perhaps because of the type of surgical procedure performed (laparoscopy) instead of a hysterectomy. Internal endometriosis or adenomyosis is a pathology of the uterine muscular wall. It is a very common benign condition, which affects women in their (3rd) 5th decade. It affects 5% to 70% depending on the series with an average close to 20% based on the analysis of hysterectomy specimens, but its exact prevalence is difficult to establish due to the variability of the diagnostic criteria sought by anatomopathologists and the absence of specific symptoms.^22^ Adenomyosis is rarely diagnosed before surgery because it causes nonspecific symptoms and clinical signs; thus, adenomyosis is most often discovered on a hysterectomy specimen.^23^

Benign tumors represented the most frequent pathologies 39.0% (2599). Among these tumors, leiomyomas were 29.6% (1954), and there was a significant association between endometriosis and leiomyoma (OR >1; *p* < 0.05). The rate of coexistence of endometriosis and leiomyoma remains poorly understood, as few studies have investigated the association.^24,25^ Both contribute to considerable pains and can lead to subfertility or infertility in women.

A study conducted in California between 2011 and 2015, in which most women were black, demonstrated that patients who have symptomatic leiomyoma may also be at higher risk of endometriosis.^26^ The authors of this study highlighted the importance of maintaining a high level of suspicion of endometriosis before and during surgery in these women, with the aim of treating both pathologies in a single surgery.^26^

Salpingitis was a factor favoring the occurrence of endometriosis (OR = 1.9; *p* < 0.0001). It had been demonstrated that isthmic salpingitis nodosa is associated with infertility and the occurrence of tubal pregnancy.^27,28^ Antibiotic therapy makes the signs of salpingitis disappear, but the anatomical changes that these causes can persist and therefore induce ectopic pregnancies. This perhaps explains the presence of associations between endometriosis and salpingitis, on the one hand and on the other hand, the association between endometriosis and ectopic pregnancies observed in women living in Gabon during this our study.

The logistic regression showed that age, in particular the groups of [36–50] years and [51–89] years, salpingitis, and benign tumors were risk factors for the occurrence of endometriosis. It could be explained by the pathophysiology of endometriosis in relation to menstrual cycles which are quantitatively more important in these age groups.^20^ In addition, it is easier to perform surgery at ages around menopause, after 40 years old, even more, so to rid/relieve the patient of a uterus causing her pains.

As a retrospective study, this study presented some limitations linked to information biases (absence of information on parity, gestational age, reasons or symptoms which prompted the surgeries, *etc.*). These aspects will be the subject of a subsequent investigation.

The lack of cases of endometriosis at the thoracic level during this study does not mean that there were none. This could simply be due to the fact that the study did not include surgical parts samples from the thoracic part of women. The thoracic form of endometriosis is increasingly common,^29^ but it has never been described in Gabon because until now, no study has focused on endometriosis in this country. This absence could also be linked to a lack of specialists in thoracic surgery. However, the lack of thorax parts represents a major bias in both selection and information. In sub-Saharan Africa, only a few studies have been published on thoracic endometriosis.^29^

Given the many associated pathologies and lesions, it seems that most endometriosis diagnoses were incidental findings. The average age of women with endometriosis was greater than that of women in general during this study, suggesting a late diagnosis. It seems that the adenomyosis form was the most diagnosed. The age range of women [56–68 years] is supposed to contain predominantly menopausal women, yet it accounted for 5% of cases of endometriosis. Hence, the need for a prospective study to better analyze endometriosis in the Gabonese context. Do all women with endometriosis come to the hospital, or do some visit traditional therapists?

## Data Availability

The datasets used and/or analyzed during the current study available from the corresponding author on reasonable request.

## Abbreviations used

KAP: Knowlege Attitude and Practice
LAP: The laboratory of the Department of Pathological Anatomy
USS: The University of Health Sciences
